# Elemental Silver Nanoparticles: Biosynthesis and Bio Applications

**DOI:** 10.3390/ma12193177

**Published:** 2019-09-27

**Authors:** Oleg V. Mikhailov, Ekaterina O. Mikhailova

**Affiliations:** Analytical Chemistry, Certification and Quality Management, Kazan National Research Technological University, K. Marx Street 68, 420015 Kazan, Russia; katyushka.glukhova@gmail.com

**Keywords:** Ag-NP, geometric shape, green synthesis, plants, microorganisms, biological applications

## Abstract

The data on the specifics of synthesis of elemental silver nanoparticles (Ag-NP) having various geometric shapes (pseudo spherical, prismatic, cubic, trigonal-pyramidal, etc.), obtained by using various biological methods, and their use in biology and medicine have been systematized and generalized. The review covers mainly publications published in the current 21st century. Bibliography: 262 references.

## 1. Introduction

Elemental silver nanoparticles (Ag-NP) have been known in anthropogenic activities since very ancient times (although nobody had an idea about their existence). For example, detailed study of one of the late Roman Empire cultural masterpieces, namely the Lycurgus Cup (IV century ad), has shown that the glass inserts in its bronze frame owe their specific coloring (red in reflected light and gray-green in transmitted) to the presence of nanoparticles that are 70% elemental silver [1]. Even earlier (II century ad), so-called “Holy Water”, which is not exposed to infection by microorganisms and spoilage for many months and years, was known; moreover, it has a very detrimental effect on a wide variety of pathogenic microorganisms [2]. At the end of the 19th century, the phenomenon “oligodynamia”—the silver bactericidal effect on the cells of microorganisms by Ag^+^ ions—was found by the Swiss botanist K. Nägeli [3]. However, more detailed study about the antibacterial activity of “Holy Water”, carried out in the second half of the 20th century, showed that it is connected with both the presence of Ag^+^ ions and the presence of Ag-NP [4]. Herewith, among elemental metal nanoparticles, namely elemental silver has the strongest bactericidal effect [5,6], which is a direct consequence of the optimal ratio of their surface areas and volumes. Now the bactericidal, bacteriostatic, antiviral, antifungal and antiseptic effect of silver ions and Ag-NP suspensions has been shown on more than 500 pathogenic microorganisms, yeast fungi and viruses. Moreover, their antibacterial and antiviral activity is even more pronounced than the effect of penicillin, biomycin and other “classic” antibiotics [7,8]. Low probability of certain mutations with the result of resistance to Ag-NP becomes extremely important in the struggle of microbiologists with an ever-growing assortment of pathogenic bacteria and viruses that are resistant to traditional antibiotics. This important circumstance, relatively low toxicity and allergenicity of Ag-NP, and its good tolerance by patients, has contributed to the increased interest in Ag-NP in many countries of the world and the creation of various medical preparations based on anti-inflammatory, antiseptic and bactericidal action.

The study of the mechanism of antibacterial activity of Ag-NP showed that this property is due to morphological and structural changes in bacterial cells [4]. A priori, it is obvious that the degree of this effect should depend significantly on the size and shape of Ag-NP. On the other hand, in many studies of recent decades, the size, morphology, stability, and both chemical and physical properties of elemental metal nanoparticles, including silver, were very dependent on the parameters of the processes by which they are formed. These parameters are directly related to the specifics of the given processes, reactions of the metal ions interaction with reducing agents, and sorption processes of stabilizing agents on nanoparticles, which prevent their aggregation with each other [9]. In general, control of the shape, size and distribution of the resulting Ag-NP is achieved by varying the methods of their synthesis, reducing the influence of fluctuations, and stabilizing factors affecting the above parameters of nanoparticles [10,11,12,13,14]. By varying the conditions for the synthesis of Ag-NP, characteristics such as color, melting point, magnetic properties, redox potential of Ag(I)/Ag, etc. can be changed and controlled in a fairly wide range [15,16,17,18,19,20]. Comprehensive coverage of Ag-NP problems was presented in reviews [19,20]. However, the main focus was on the synthesis of Ag-NP using various physicochemical methods. Another synthetic method, in which some objects of biological origin are used to obtain elemental silver nanoparticles, has become increasingly popular. This approach has certain advantages in comparison with traditional physicochemical methods, and the possibilities of its implementation for the production of Ag-NP are not only not exhausted, but even not completely identified. The review is dedicated to this issue. 

## 2. General Principles of Biosynthesis of Elemental Silver Nanoparticles (Ag-NP)

The basis of all chemical and physicochemical methods for the synthesis of Ag-NP is the idea of a specific increase in the area of their faces in the presence of certain chemical reagents. Biological methods base on similar idea, but biological objects (microorganisms, products of their vital activity, extracts of plants, etc.) are used for the formation of Ag-NP. As a rule, Ag-NP is obtained as a result of the reduction of certain Ag(I) compounds (usually AgNO_3_), under the influence of various reducing inorganic agents (f.e., hydrazine N_2_H_4_, sodium tetrahydridoborate(III) Na[BH_4_]), as well as organic (f.e, ethyleneglycol, ascorbic acid). The process of reducing Ag(I) compounds to elemental silver according to the Ag(I)→Ag scheme can occur in a variety of reaction media; at the same time, specific chemical reagents (most often polyvinylpyrrolidone) are used to stabilize the resulting nanoparticles (to prevent their aggregation). The formation of Ag-NP, according to data [19,21,22], begins with the incipience of a decahedral “embryo” formed by five tetrahedral clusters that have common faces. Since the dihedral angle in the tetrahedron is ~70.5°, namely the decahedron, consisting of five tetrahedrons, is that structural element from which the most thermodynamically stable forms of Ag-NP. It should be noted that the dihedral angle in the tetrahedron indicated above is slightly smaller than the angle in the above decahedron (72°); therefore, tetrahedrons cannot be perfectly packed into a decahedron without gaps (which lead to the formation of structural defects) [19]. Such a scheme displays only a simplest variant of the formation of Ag-NP, which leads to the formation of elemental silver nanocrystals in the form of nanorods or “nanowires”. In reality, this process often turns out to be significantly more complex and interesting, and as a result, other geometric forms—tabular prisms, cubes, octahedrons, pyramids etc., can be realized [19,20]. As it was shown in various studies, the shape and size of the resulting Ag-NP depend on many experimental parameters. These can be the temperature, the concentration of the compound Ag(I), the pH of the solution, the molar ratio between stabilizing agent and the precursor containing Ag(I) (AgNO_3_, AgCl, et al.), the degree of chemical interaction of the molecules of the stabilizing agent with various crystallographic planes of elemental silver [22]. The nature of the reducing agent of Ag(I) compounds (citric acid, *L*-ascorbic acid, NaBH_4_ et al.) and the method used to produce Ag-NP (chemical, physical or biological) play an extremely important role, too. Using particles with a specific morphology (for example, polyhedral or twin) as a “primer”, it is possible under certain synthesis conditions to purposefully control the final “architecture” of Ag-NP [19,23,24].

The chemical and physicochemical methods used to produce Ag-NP, are usually quite expensive; in addition, toxic chemical compounds are often used in them. Biological methods are practically devoid of these disadvantages. Of course, they are also not ideal: it should be noted that the reproducibility of the Ag-NP synthesis results is worse than in the case of physicochemical methods. Despite this, the number of works about Ag-NP biosynthesis has a pronounced tendency to annual growth. In modern literature, Ag-NP biosynthesis is often called “green synthesis”.

The three key types of biological objects used for this purpose–extracts of various plants, various microorganisms, and animal products, can be distinguished in the literature about Ag-NP biosynthesis. It should be noted that many works devoted to this problem, were published either in biological journals or in medical journals. Most of these publications were devoted not only to Ag-NP biosynthesis, but also the possibility of using silver nanoparticles as antibacterial agents. This aspect of Ag-NP characterization of, of course, is very important and interesting, and it will also be analyzed in this review along with a discussion of their synthesis.

Now there are a lot of papers in the field of the Ag-NP “green synthesis”. In this regard, a difficult question arises as to how to systematize the available material on the given problem. The “catchiest” characteristic of nanoparticles and Ag-NP, undoubtedly, is their shape, because it largely determines their properties (including the properties of the materials in composition of which are these nanoparticles). For this reason, available literary material was systematized by the types of biological objects used for the synthesis of Ag-NP and by the shape of elemental silver nanoparticles formed in this process.

Getting a little ahead in the course of the presentation, we would like to note that such geometric shapes of Ag-NP, which were obtained using chemical and physicochemical methods, apparently, could also be obtained using biological methods, although not all of these possible shapes were received experimentally.

## 3. Synthesis of Ag-NP Using Extracts of Various Plants

Plant extracts obtained from leaves, stems, roots, etc. as the result of exposure to various liquid solvents—extractants. Water, ethanol, dimethyl ether, plant oil, etc. can act as extractants. Plant extracts are complex in composition, containing various chemical compounds arising in the process of plant life, and transferring into solution during extraction. In the process of the synthesis of Ag-NP according to the general scheme Ag(I)→Ag, the chemical compounds contained in the extract can fulfill three functions: (1) act as a reducing agent of silver(I) compounds to elemental silver, (2) act as an agent that has a specific influence on the formation of a certain shape and size of Ag-NP due to inhibition of the growth of certain faces of the nanocrystals of these nanoparticles and (3) to act as a stabilizer formed during the synthesis of Ag-NP, preventing their self-association and (or) aggregation with each other. Due to the fact that the composition of the original plant material depends significantly on the type of a particular plant, the qualitative and quantitative composition of the resulting extracts can vary widely. Moreover, even for the same plant species, it does not remain constant and in some cases depends significantly on the conditions of its growth. Therefore, it is likely that the technology of synthesis of Ag-NP using extracts of the same plant, but in laboratories located in different regions of our planet, can lead to very different final results (we mean the shape and size elemental silver nanoparticles). Thus, the reproducibility of Ag-NP biosynthesis results will not be too good a priori. It should be noted that despite the very large number of works devoted to the Ag-NP biosynthesis using plant extracts, among them there are not even two such works in which extracts from the same plant species were used, but grown in different geographical, climatic and soil conditions.

**Figure 1:**
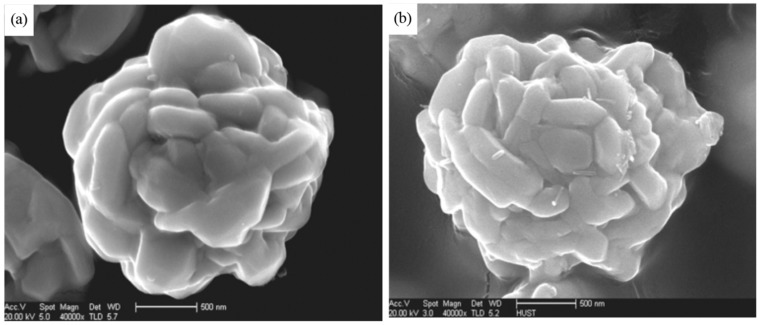
Typical scanning electron microscope (SEM) images of pseudo-spherical elemental silver nanoparticles (Ag-NP) at high resolution (**a**) none modifier; (**b**) at the presence of 1.0 g/L N-methyl 2-pyrrolidone [24].

An extremely large number of publications have been devoted to the “green synthesis” of Ag-NP using biological objects of this type [25,26,27,28,29,30,31,32,33,34,35,36,37,38,39,40,41,42,43,44,45,46,47,48,49,50,51,52,53,54,55,56,57,58,59,60,61,62,63,64,65,66,67,68,69,70,71,72,73,74,75,76,77,78,79,80,81,82,83,84,85,86,87,88,89,90,91,92,93,94,95,96,97,98,99,100,101,102,103,104,105,106,107,108]. In all of them only AgNO_3_ was used as a precursor containing Ag(I) for the Ag-NP synthesis. Particles having a spherical and/or oval (ellipsoidal) shape were identified in most of the experiments; shapes differed from those, were observed in the experiment much rarely [91,92,93,94,95,96,97,98,99,100,101,102,103,104,105,106,107,108]. In this connection, it should be noted that, spherical and oval shapes are conglomerates of smaller “embryonic” particles of elemental silver, which are complex combinations of “starting” geometric forms indicated in [19]. To some extent, this can be confirmed by scanning electron microscope (SEM) images of spherical Ag-NPs at high resolution [24], shown in Figure 1. Given this fact, it would be better to call such Ag-NP pseudospherical nanocrystals. Despite this, we will continue to use the generally accepted term to refer to these objects (i.e., spherical Ag-NPs).

**Figure 2:**
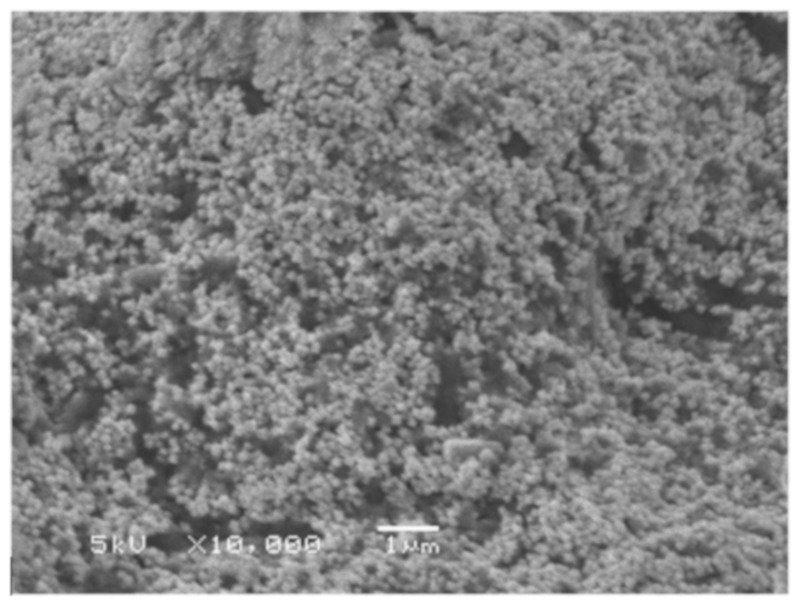
Scanning electron microscope (SEM) images of Ag-NP obtained using aqueous peel extract of *Punica granatum* [86].

**Figure 3:**
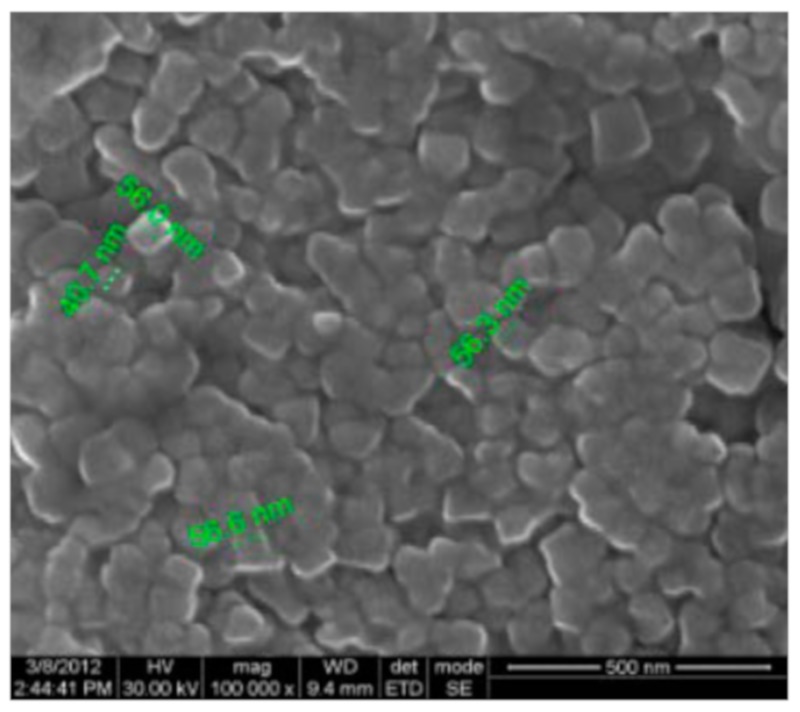
SEM images of Ag-NP obtained using aqueous extract of marine seaweed *Ulva lactucain* [42].

Among the earliest works of the 21st century devoted to the production of silver nanoparticles using plant extracts is the publication of Shankar, Ahmad and Sastry [25], in which Geranium *Pelargonium graveolens* leaf extract was used to synthesize Ag-NP. The spherical Ag-NP particles were obtained with the size varied in the range of 16–40 nm. Later, a group of researchers [26] received spherical silver nanoparticles using *Emblica officinalis* (amla, Indian Gooseberry) fruit extract with sizes from 15 to 25 nm, and, also, elemental gold nanoparticles with slightly smaller (10–20 nm) sizes. Similar results were achieved by Chandran et al [27], in which Aloe Vera leaf extract was used to synthesize Ag-NP. The spherical Ag-NP particles, the size of which varied in the range (15.2 + 4.2) nm, were shown. The authors of [28] obtained spherical silver nanoparticles using *Capsicum annuum L*. extract with sizes from 50 to 70 nm. The results the given work allow to affirm that silver nanoparticles synthesized from such a method, show antibacterial activity against *E. coli.* Cruz et al. [29] obtained a spherical Ag-NP with an average diameter of 15–30 nm, using leaf extract of *Lippia citriodora* (Lemon Verbena). Close-sized Ag-NPs were received in the work [30] using the leaf extract of *Acalypha indica*. Spherical Ag-NPs were also shown by the authors of [31] using an extract of the dried leaves of the plant *Tribulus terrestris.* The sizes of the Ag-NPs synthesized by them were in the range of (18–47) nm. The Ag-NPs obtained retained high stability (i.e., did not aggregate with each other) even after three months of storage at 37 °C. Along with this, Ag-NP received in [31] showed a pronounced antibacterial effect on a number of clinically isolated microorganisms, which have now developed resistance to many drugs. Ag-NPs of similar shape from the leaf extract of *Mimusops elengi*, were described by Prakash with co-authors in [32], but these silver nanoparticles had diameters in the range of 55 to 83 nm. In [33], spherical Ag-NPs 25–59 nm in size were synthesized using the *Chrysanthemum indicum* flower extract. In all, in the last 10 years a variety of plants were used to synthesize spherical Ag-NPs [34,35,36,37,38,39,40,41,42,43,44,45,46,47,48,49,50,51,52,53,54,55,56,57,58,59,60,61,62,63,64,65,66,67,68,69,70,71,72,73,74,75,76,77,78,79,80,81,82,83,84,85,86,87,88,89,90,91,92,93]; the information about these plants, as well as the shape and size obtained with their use of elemental silver nanoparticles are presented in Table 1 at the end of this section of the paper. As can be seen from the data in the given table, during the synthesis of Ag-NP leaf extracts were usually preferred, although in some cases other parts of the corresponding plants—flowers, roots, fruits, etc. were used for this purpose, as it took place in particular in [33,41,43,67,75,79,86,87]. Scanning electron microscopy (SEM) of Ag-NP, obtained in the one of these works [86], is presented on Figure 2. A few exceptions are publications [42,57,65], in which seaweed *Ulva lactucain* (Figure 3) [42], *Sargassum wightii* [57] and *Sargassum vulgare* [65] were used as an accompanying agent for “green synthesis” of Ag-NP. It is significant that, the synthesis of silver nanoparticles in these works was carried out, as a rule, in a neutral medium. The Ag-NP size was very diverse among using plant extracts (Table 1). At the same time, however, the question of the amount effect of plant extract used in the synthesis on the size of Ag-NP is rarely considered. One of the few such works is [58], which examines *Olax scandens.*

Leaf extract was used in the synthesis process. According to the data of this work, the average size of Ag-NP is 30–60 nm; however, the situation is rather more difficult: when the volume ratio of the AgNO_3_ solutions used by them and the extract was 1:1, the size of Ag-NP was in the range of 20–110 nm at ratios of 1:2.67 and 1:3.33—from 10 to 85 nm, with a ratio of 1:5—from 10 to 90 nm [58]. Presumably the same phenomenon would have occurred with the use of other plant extracts described in [25,26,27,28,29,30,31,32,33,34,35,36,37,38,39,40,41,42,43,44,45,46,47,48,49,50,51,52,53,54,55,56,57,58,59,60,61,62,63,64,65,66,67,68,69,70,71,72,73,74,75,76,77,78,79,80,81,82,83,84,85,86,87,88,89,90,91,92,93,94,95,96,97,98,99,100,101,102,103,104,105,106,107,108], but this point remained out of the field of view of their authors.

Ag-NPs from plant extracts with a shape different from spherical, were noted in a relatively small number of published works. In [34,41,94], the synthesis of trigonal Ag NPs having the shape of triangular plates was described. Hexagonal and spherical shapes of Ag-NPs having a size of (121 ± 2) nm were described by Arokiyaraj et al, using water extracts of *Rheum palmatum* roots [95]. The synthesized nanoparticles showed very high antibacterial activity against some pathogenic microorganisms: *Staphylococcus* and *Pseudomonas*. Using a leaf extract of *Alysicarpus monilifer*, the authors of [96] could obtain monodisperse Ag-NPs of predominantly spherical shape with a small hexagonal distortion with sizes in the range (5–45) nm and average particle size (15 ± 2) nm. At the same time, along with spherical Ag-NPs, they discovered the formation of a certain amount of Ag-NPs having a trigonal tabular and hexagonal tabular form. A small amount of the mixed phase containing hexagonal Ag-NP was also noted in an earlier article [28].

**Figure 4:**
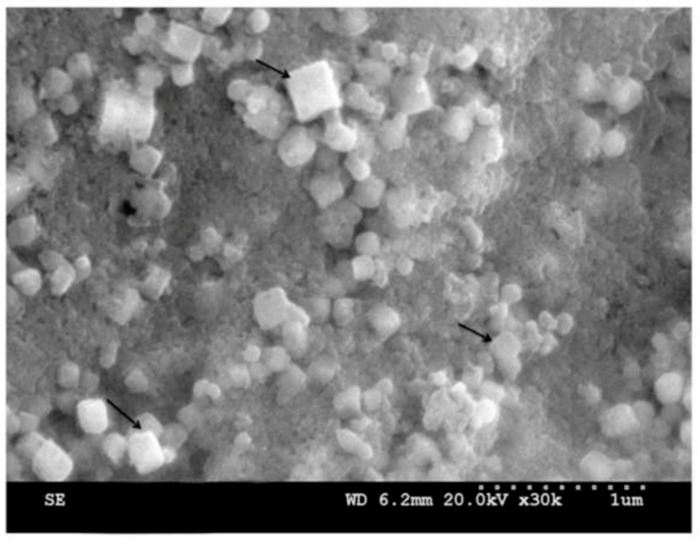
SEM images of Ag-NP obtained using aqueous leaf extract of *Melia azedarach* [97].

Ag-NP with a cubic form from plant extracts was also known in [97,98,99]. In the first of these publications, elemental silver nanoparticles were prepared using a leaf extract of *Melia azedarach*. The cubic Ag-NPs synthesized in [97] had a size of about 80 nm (Figure 4). The authors of the publication [98] reported “green synthesis” of Ag-NP, spherical and cubic shape, using leaf extracts from *Eucalyptus macrocarpa* and carried out at room temperature. During their experiment, it was also found that in the process of synthesis, this extract acts both as a reducing and stabilizing agent. Herewith, according to transmission electron microscope (TEM) data, the size of spherical Ag-NPs was in the range (10–100) nm, while the size of cubic Ag-NPs was in the range (10–50) nm. In this connection, we should note that the three-dimensional field-emission SEM (FESEM) image obtained several hours after the completion of the experiment showed that namely cubic but not spherical nanoparticles with sizes from 50 to 100 nm became the dominant shapes [98]. In [99], three different plants were used to receive such cubic nanoparticles, namely *Cucurbita maxima*, *Moringa oleifera*, and *Acorus calamus*. In this regard, it is interesting that various parts of these plants (peels, leaves and rhizome, respectively) were used to prepare plant extracts. The sizes of silver nanoparticles obtained in [99] varied in the range of 30–70 nm.

**Figure 5:**
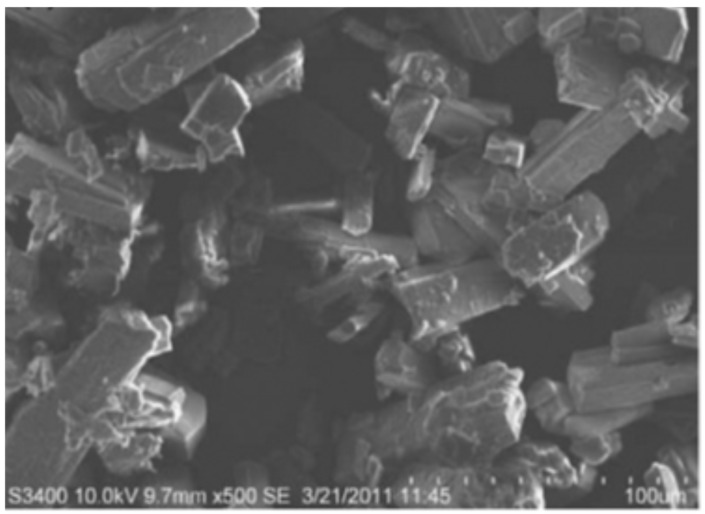
SEM images of Ag-NP obtained using aqueous leaf extract of *Syzygium cumini* [100].

**Figure 6:**
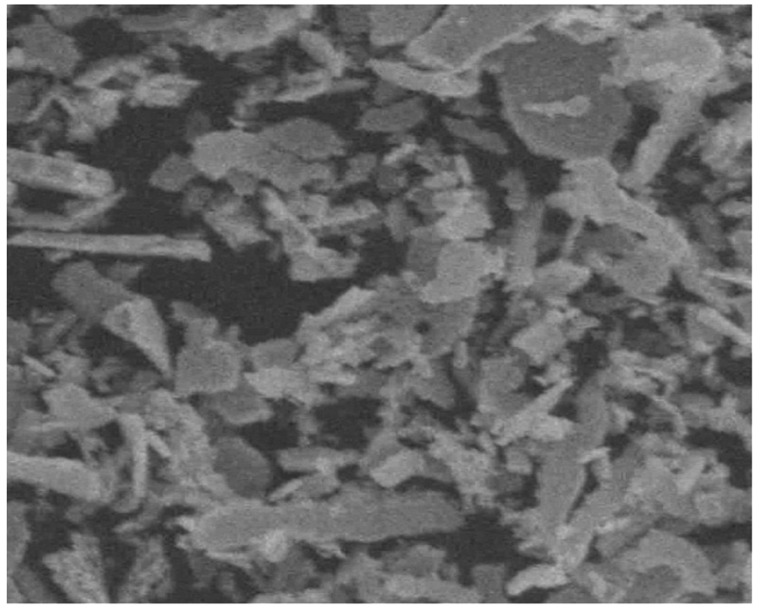
SEM images of Ag-NP obtained using aqueous leaf extract of *Artemisia nilagirica* [105].

The authors of [100] observed the formation of prismatic Ag-NPs with sizes of 22–65 nm using leaf extracts of *Ocimum tenuiflorum*, *Solanum tricobatum, Syzygium cumini, Centella asiatica*, and *Citrus sinensis* for “green synthesis”. For these Ag-NPs, a strong tendency toward coalescence was found, which is most represented in the case of *Centella asiatica* and least in the case of *Syzygium cumini* (Figure 5). Baharara et al. in the publication [101] described the synthesis of elemental silver nanoparticles, some of which had a pentagonal-tabular shape; in the absorption spectra of these Ag-NPs, a distinct maximum was observed at 450 nm [101]. Along with this, in a number of works on biosynthesis using plant extracts [102,103,104,105,106,107], other (mainly irregular) forms of Ag-NP were noted. So, in [105] by using leaf extract *Artemisia nilagirica,* silver nanoparticles shown in Figure 6 were synthesized. As can be seen, they are shapeless conglomerates and blocks resembling a pile of stones.

From the extract of the leaves of *Tinospora cordifolia* [106] Ag-NP particles were obtained with an external form that resembles a highly distorted sphere, and with the use of extracts of *Leucas aspera* and leaves of *Hyptis suaveolens* particles, some of which had a distorted spherical shape, and other polygonal shape were shown [107].

The completely unusual “flower-like” form of Ag-NP was observed by Pourjavadi and Soleyman in [108]. In their study, with the remarkable name “Novel silver nano-wedges for killing microorganisms”, a peculiar photochemical surface “green synthesis” was first applied to produce Ag-NP using the “Salep” (tuber extract of *Orchis mascula*). In the framework of such a synthesis, the reduction of Ag(I) to Ag-NP, in addition to the extract itself, was facilitated by sunlight (ultraviolet (UV) radiation). “Salep” also served as an effective capping biomaterial, providing the formation of “flower-like” self-organizing structures in the form of unique silver “nano-wedges”. In addition, there was an aggregation of such structures, which resulted in the formation of Ag-NPs with the above unusual shape. 

As can be seen from the data presented in Table 1, in most cases for Ag-NP synthesized using plant extracts, in the visible region of the spectrum, either a single absorption band with a maximum in the range of 400–460 nm or a “wing” band with a maximum in the Ultra-Violet region (UV) region were detected. Accordingly, their colloidal solutions are usually colored in orange, red or red-brown. However, any correlation between the sizes, as well as the shape of the nanoparticles and the position of this maximum in the ultraviolet–visible (UV–Vis) absorption spectra, as can be seen from experimental data presented in the Table 1, was not observed.

Review articles [109,110,111,112,113] were also devoted to the discussion of recent results on Ag-NP biosynthesis using plant extracts, in which references to a number of other, earlier works devoted to the “green synthesis” of silver nanoparticles using extracts of various plants can be found. A possible mechanism of the plant extracts’ influence on the process of formation of Ag-NP has been considered in detail in recently published reviews [113,114].

## 4. Synthesis of Ag-NP Using Various Microorganisms

The use of microorganisms (bacteria, microscopic fungi, etc.) for the synthesis of Ag-NP according to the general scheme Ag(I)→Ag is based on the idea that they produce specific chemicals during their life, each of which can a priori perform the same functions (1–3), which were described in the previous section. In addition, these microorganisms are able to affect a certain influence on the size and shape of the elemental silver nanoparticles formed by themselves (i.e., outside of the connection with the nature of those products that are formed in the process of their development). In particular, in the final period of the formation of Ag-NP, when these nanoparticles will have reached relatively large sizes, microorganisms can overlap, with some fragments of their bodies, individual faces of already formed elemental silver nanocrystals, preventing their further growth. Thus, the action of microorganisms on the process under consideration is more multifactorial than the impact of plant extracts. However, in this variant of Ag-NP biosynthesis, the reproducibility of the results should be higher, because modern biotechnologies make it possible to purposefully control both the growth of microorganisms and the various substances produced by them.

The specificity of Ag-NP biosynthesis using various microorganisms was studied in the works [115,116,117,118,119,120,121,122,123,124,125,126,127,128,129,130,131,132,133,134,135,136,137,138,139,140,141,142,143,144,145,146,147,148,149,150,151,152,153,154,155,156,157,158,159,160,161,162,163,164,165,166,167,168,169,170,171,172,173,174,175,176,177,178,179,180,181,182,183,184,185,186]. In most of these publications, various bacteria were used as biological objects [115,116,122,123,124,125,127,128,130,132,133,134,135,136,138,140,141,143,144,146,147,150,151,153,154,155,156,158,161,163,164,165,166,169,171,172,173,174,175,176,177,178,179,180,183,184,185,186]; rare such synthesis was carried out with the participation of various microscopic fungi [117,118,119,120,121,126,129,131,137,139,142,145,148,149,152,157,162,167,168,170,181,182]. It should be noted, as well as in the case of using plant extracts, the formation of pseudospherical Ag-NP most often took place [115,116,117,118,119,120,121,122,123,124,125,126,127,128,129,130,131,132,133,134,135,136,137,138,139,140,141,142,143,144,145,146,147,148,149,150,151,152,153,154,155,156,157,158,159,160,161,162,163,164,165,166]; other forms of silver nanoparticles, although noted in the experiment, were much rarer [115,116,132,133,167,168,169,170,171,172,173,174,175,176,177,178,179,180,181,182,183,184,185,186]. Data on the size and shape of Ag-NP obtained using various microorganisms are presented in Table 2. 

**Figure 7:**
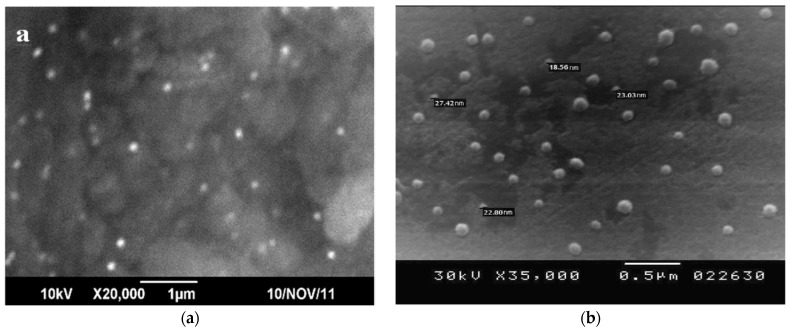
SEM of spherical Ag-NP obtained in [147] (**a**) and obtained in [155] (**b**).

One of the earliest works devoted to the biosynthesis of Ag-NP using microorganisms are the publications [115,116,117,118], where, along with spherical Ag-NP, nanoparticles with a different shape, namely, triangular and hexagonal, were described. After that, a lot of works on this topic appeared. The most popular microorganisms in the “green synthesis” of elemental silver nanoparticles are bacteria of the genus *Bacillus* [122,123,130,132,133,140,143,154,159,169,179,180,183]. So, the authors of [122], using *Bacillus licheniformis*, obtained pseudospherical Ag-NP with an average size of about 40 nm, in the visible spectrum of which there was only a “wing” of the absorption band with a maximum in the UV region. The same kind of microorganisms was also used by Kalimuthu with co-authors [123], who, under somewhat different experimental conditions, showed pseudospherical Ag-NP with an average size of about 50 nm, in the visible spectrum of which there was a band with a peak at 440 nm. Saravanan et al. [159] synthesized Ag-NP having a size range of 41–68 nm with a spherical shape using *B. brevis* (NCIM 2533). Similar results were received using *B. megaterium* (NCIM 2326) for the synthesis of Ag-NP in another work of this author [169]. Overall, in most cases, only pseudospherical nanoparticles were the final products of biosynthesis using microorganisms. However, in some of the works, the researchers also recorded the formation of silver nanoparticles with other external shapes (Table 2). For example, using *B. subtilis* and *B. amyloliquefaciens*, the authors [132] and [133] respectively were able to observe the formation of triangular and hexagonal silver nanoparticles, along with spherical Ag-NP, and triangular, hexagonal and cubic shapes were described in [180]. The sizes of Ag-NP obtained in this case ranged from 2 nm in [180] to 99 nm in [169] (Table 2). It should be noted in this connection that triangular and hexagonal Ag-NP, using not these bacteria themselves (in this case, *B. licheniformis*), but the isolated enzyme from them, α-amylase, was received by Mishra and Sardar [183]; the silver nanoparticles obtained had a size from 22 to 44 nm. Bacteria of other genera were used for the biosynthesis of Ag-NP as a whole much rare, although in general their assortment is quite large (Table 2). Most often pseudo-spherical Ag-NP with very diverse sizes, very significantly depending on the nature of the microorganism, were shown. Examples of Ag-NP images with such an external shape received using bacteria are shown in Figure 7. It should be noted that any correlation (at least in qualitative terms) between the genus of bacteria and the parameters of those nanoparticles of elemental silver (size and shape of Ag-NP), which are formed with the active participation of these microorganisms, has not yet been detected.

**Figure 8:**
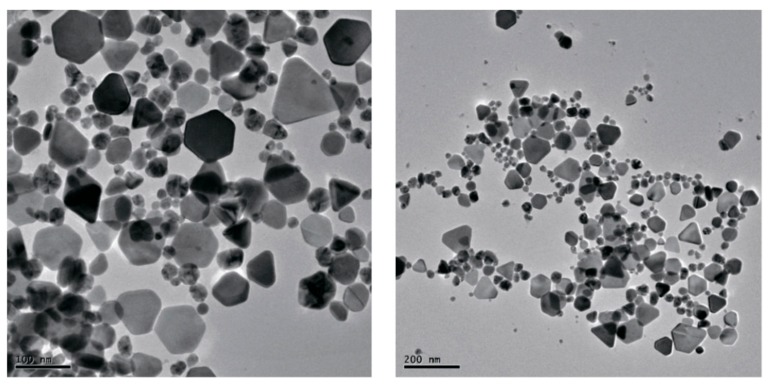
Transmission electron microscope (TEM) image of Ag-NP having various shapes and obtained in [186]. At a scale of 100 nm (**left**) and at a scale 200 nm (**right**)

**Figure 9:**
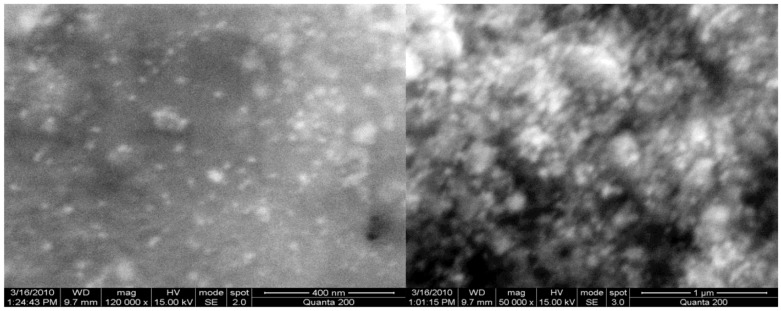
SEM image of Ag-NP having irregular shape and obtained in [176]. At a scale of 400 nm (**left**) and at a scale 1 m (**right**).

Very interesting results were presented in an article by Husain, Sardar, and Fatma [185], in which the authors studied the possibilities of “green synthesis” of Ag-NP using various cyanobacteria. According to the data presented in this publication, out of 30 microorganisms used for the biosynthesis of Ag-NP, and in nine cases, nanoparticles with shapes other than spherical, and, namely, triangular, pentagonal, hexagonal and cubic were described (Table 2). In the spectrum of each Ag-NP showed in [185], there was one absorption band in the violet, blue, or blue region of the visible spectrum with λ_max_ in the range 440–490 nm. A larger assortment of elemental silver nanoparticle forms was noted by P. Singh et al. in [186], where in addition to the triangular, pentagonal, hexagonal and cubic already mentioned above, icosahedral and truncated triangle shapes were presented. In addition, that it is interesting, in this work, such diversity was achieved using only ONE microorganism: *Bhargavaea indica* (Figure 8). Despite such a considerable variation in the shape, as well as in size (30–100 nm) of Ag-NP obtained in [186], the authors of this work noted the presence of a band with only one λ_max_ value, namely 460 nm, in all these nanoparticles. (Although there are strong reasons to believe that Ag-NPs of different shapes should have λ_max_ values that are different from each other). In some publications on the biosynthesis of Ag-NP using various microorganisms [169,171,172,173,174,175,176,177,178,179], the formation of nanoparticles with irregular shape was also noted (Figure 9).

Along with microorganisms microscopic fungi were used as substrates for AgNP biosynthesis. First of all, it was fungi of the genus *Aspergillis* [120,126,139,167,168,170,182], *Penicillium* [131,142,145,152,162] and *Fusarium* [118,119,121,148,181]. This “green synthesis” of Ag-NP, as a rule, also led to the formation of pseudo-spherical Ag-NP with a wide variety of sizes (Table 2). So, in [118], as well as in [119], Ag-NP with such an external shape were obtained using *Fusarium oxysporum* fungi, and in the first of these works, silver nanoparticles were noticeably smaller than in the second (5–15 nm and 20–50 nm, respectively). Micro-fungi *F. semitectum*, were used as a substrate by the authors [181]; besides, Ag-NP with sizes in the range of 10–60 nm were received. Using fungi of the genus *Aspergillis* elemental silver nanoparticles with sizes ranging from 3 nm [126] to 140 nm [139] were described. The same situation took place for Ag-NP with using fungi of the genus *Penicillium*, but the size range was much smaller—from 8 nm [131] to 50 nm [152] (Table 2). Mostly get Ag-NP with the use of micro-fungi were also pseudo-spherical, but in some cases also took place the formation of small quantities of silver nanoparticles different geometric shapes [167,168,170,181,182]. For example, the authors of [181], along with pseudospherical Ag-NP, also obtained silver nanoparticles having a hexagonal shape. The formation of elemental silver nanoparticles having an irregular shape were observed in [167,170]. (An irregular shape of Ag-NP was also described by the authors [168] with a size of 550–650 nm, which although it refers to AGP aggregates, goes beyond the range accepted for nanoparticles (1–100) nm).

As with the use of plant extracts in Ag-NP biosynthesis, in most cases, for elemental silver nanoparticles synthesized using microorganisms, either a single absorption band with a maximum in the range of 400–460 nm in the visible spectral region or a “wing” band with a maximum in the UV region were also detected. Their colloidal solutions are also colored either in orange, or in red, or in red-brown. As an exception, only Ag-NP obtained by Manikprabhu and Lingappa in [173] using bacteria of the genus *Staphylococcus*, for which authors found the presence of two bands in the visible region of the spectrum, namely, violet with λ_max_ in the range 420–430 nm and in yellow-green with λ_max_ in the range of 550–570 nm was presented. However, the second of these bands was weakly expressed, as a result of which the Ag-NP obtained in [173] in color did not stand out among the other Ag-NP received with the use of microorganisms. These nanoparticles had sizes ranging from 28 to 50 nm and an irregular shape [173].

Concluding this section, we note that during the biosynthesis of Ag-NP using both plant extracts and microorganisms, it has not yet been possible to obtain such shapes of silver nanoparticles as nanorods, nanowires, or nanobars, which were once observed during physicochemical synthesis of Ag-NP, in particular, in the publications [187,188,189]. During this biosynthesis, Ag-NP particles with that unique “flower-like” shape that was described in the work cited above [109] have also not yet been discovered. On the biosynthesis of Ag-NP using various microorganisms, there is, in particular, a review article [114].

## 5. Synthesis of Ag-NP Using Various Protein Products

The literature contains a number of data showing the possibility of using for the synthesis of Ag-NP various products of animal origin, in particular polypeptide high molecular weight compounds (proteins). Owing to the large size of their molecules, the molecular mass of which (M) is tens and hundreds of thousands of carbon units (c.u., Daltons), their role in this process is reduced mainly to function (3), i.e. substrates that hinder the aggregation of already formed nanoparticles (although in principle their participation in the functions referred to in paragraph III (1–2) is not excluded); in that way, these nanoparticles are immobilized in masses of these substrates. An example of such a substrate is gelatin, which is the main component of various food (in particular, meat) jellies. As known [190,191,192,193,194], this natural compound is a polydisperse mixture of low molecular (molecular weight M = 50,000–70,000 c.u.) and high molecular (M = 200,000–300,000 c.u.) polypeptides. The dimensional structure, which is now well studied [195,196,197], and in this structure there are many cavities of nanoscale size, which can serve as a kind of molecular nanoreactors. The reduction reaction Ag(I)→Ag occurs namely in such cavities; in this case, some water-insoluble silver compound (AgCl, AgBr, Ag_4_[Fe(CN)_6_] et al) immobilized in a gelatin mass acts as a silver-containing precursor. The reducing agent is an organic or inorganic substance with strongly pronounced electron-donor properties. One of the most suitable for this purpose is tin dichloride SnCl_2_, which was used to obtain gelatin-immobilized Ag-NP in [198,199,200,201]. The reduction according to the Ag(I)→Ag scheme occurred in a strongly alkaline (pH~12) medium in the presence of reagents capable of forming fairly strong and water-soluble complexes with Ag(I) (thiocyanate anion SCN^−^, thiosulfate anion S_2_O_3_^2−^, ammonia NH_3_, ethylenediamine, monoethanolamine, etc.). Due to this fact, not AgCl, AgBr, or Ag_4_[Fe(CN)_6_], but Ag(I) complexes with the inorganic and organic compounds named above, were actually reduced. The size of the pseudospherical Ag-NP obtained in this variant of biosynthesis is in the range from 10 to 40 nm; this was first experimentally established in [199]. In this regard, when these nanoparticles are isolated from the gelatin matrix (for example, by the action of proteolytic enzymes, as described in [199], their aggregation naturally occurs; nevertheless, a significant part of these particles retains its former nano size. Details of the production of Ag-NP using such a specific method were presented in [200,201]. The idea that in the specific conditions of chemical processes in the gelatin matrix, as well as due to the above-mentioned specific structure of the gelatin itself, nanoparticles of a wide variety of chemical compounds can be formed in it, was expressed in a number of earlier works, particular in reviews [195,196,197,202,203].

Gelatin is not the only polypeptide substrate that can be used for this purpose; so, the various albumins are known for the synthesis of Ag-NP [204,205,206]. For example, the chicken egg protein was used for this purpose [204]; as a result, spherical Ag-NP with an average size of ~20 nm and a maximum in the visible spectral region at 425 nm were obtained. The same substrate was used in [205], and in [206]—bovine serum albumin. Some publications concerning protein- and peptide-directed syntheses of inorganic materials, and, in particular, of elemental noble metal nanoparticles having various sizes and morphologies, can be found in review [207].

Another suitable substrate for the synthesis of Ag-NP can be a natural biopolymer chitin and chitosan derived from it. Chitosan is characterized by so-called mucoadhesive properties (ability to adhere to various mucous membranes) [208], which seems to be very important for creating drugs that enter the body through the mucous membranes [209,210,211]. In this connection, it seems appropriate to obtain chitosan-immobilized Ag-NP, which could be used as effective antibacterial agents. Now, however, only fragmentary information is available [212,213,214,215,216,217,218,219,220]. The influence of chitosan molecular weight on Ag-NP dimensional characteristics when they were formed in situ as a result of reduction of AgNO_3_ precursor in solution of this biopolymer was studied by Apryatina et al. [212,213]. It is very important that the authors [212,213] were able to regulate the size of silver nanoparticles formed in the range from 8 to 12 nm by changing the molecular weight of chitosan (which also plays the role of a stabilizer of silver nanoparticles occuring during biosynthesis). The effect of the chitosan (M) molecular mass on the spectral characteristics of Ag-NP is also interesting: for example, at M = 40,000 c.u. the absorption maximum in the visible spectrum (λ_max_) is at 424 nm, at M = 127,000 c.u., at 412 nm, at M = 165,000 c.u., at 400 nm, at M = 240,000 c.u., at 383 nm. Herewith, Ag-NP formed in chitosan solutions with a higher molecular mass and having a size of 8 nm, exhibit much more pronounced bactericidal activity than Ag-NP with a size of 12 nm [212,213]. In a recently published paper [214] Uryupina with co-authors obtained pseudospherical Ag-NP with an average size of 65 nm using chitosan.

In [215,216,217,218,219], another derivative of chitin, namely 6-O-carboxymethylchitin, was tested as a substrate, and the use of γ-radiation from the ^60^Co isotope contributed to the restoration of the AgNO_3_ precursor. As a result of the studies, new radiation-induced bactericidal metal-polymer nanosystems containing the above biopolymer and elemental silver nanoparticles, were created. Besides, by varying the dose of γ-radiation, as well as the degree of filling of the biopolymer macromolecules with Ag^+^ ions, the authors of works [215,216] were able to create macromolecular systems with silver nanoparticles 1–5 nm in size and, most importantly, to control these sizes during the experiment. The Ag-NP obtained in these studies, however, had an irregular shape. Research in the field of chitosan-immobilized Ag-NP undoubtedly requires continuation.

Thus, in principle, other high-molecular compounds belonging to the number of polysaccharides, for example, agar-agar, carrageenan and guar, can be used as substrates for the production of silver nanoparticles. In [220] pseudo-spherical Ag-NP using the guar as a substrate with sizes of 10–30 nm and λ_max_ in the range of 410–425 nm were received. However, according to the authors of this article, systematic research in this direction has not yet been undertaken.

## 6. Bio Applications of Ag-NP

Currently, elemental silver nanoparticles obtained by various methods are used in a very diverse fields of science and technology. Thus, an important area of application of Ag-NP is catalysis, which can be implemented in two versions: with the influence on the reaction system of electromagnetic radiation (photocatalysis) and without it. For example, in [221,222] it was shown that Ag-NP on a SiO_2_ matrix exhibits catalytic properties in redox reactions involving benzene, carbon monoxide, some dyes, and, possibly, many other chemical compounds. In particular, benzene under these conditions is almost completely oxidized to phenol even when the Ag-NP content in the matrix is about 1 mass. %. Reactions between sodium borohydride and dyes such as methylene blue and eosin, in the presence of Ag-NP in the reaction system, proceed at a very high rate, whereas in their absence such reactions practically do not take place. Besides, the SiO_2_ substrate actually serves only to prevent the aggregation of Ag-NP in a colloidal solution [222]. An important feature of Ag-NP is that they allow photocatalysis to be realized for the creation of resonant surface plasmons from light in the visible range, as well as to enhance the fluorescence intensity [223,224,225,226,227,228]. Owing to their stability and oxidation stability, elemental silver nanoparticles are widely used, for example, in electronics and photonics [229], as a biosensor [230], in biocatalysis [231], for protein coagulation [232] and for drug delivery [233]. A layer of silver nanoparticles covered cutlery, door handles and even a keyboard and mouse for computers; they are used to create new coatings and cosmetics, in filters of air-conditioning systems, in pools, showers and other places. The method of isotropic printing for the manufacture of silver microelectrodes is described, in which samples of electronic components with a minimum width of about 2 μm were received by applying a concentrated paint consisting of silver nanoparticles on semiconductor, plastic and glass substrates [234].

The foregoing, however, relates mainly to those Ag-NP that were produced by chemical and physicochemical methods. And although Ag-NP obtained using “green synthesis” could also find their application in the above areas of science and technology, nevertheless, their modern practical application is related to the field of their production. As in this case biosynthesis of Ag-NP, as a rule, was carried out in laboratories of biological and/or biochemical section with participation of experts in the field of biochemistry and biotechnology; attention of researchers was focused on the application of Ag-NP in biology, first of all in medicine and pharmacology.

Currently, significant factual material related to bioapplications of Ag-NP already exists [235,236,237,238]. First of all, it is the possibility of antimicrobial activity of Ag-NP; according to the data presented in [7,8], their antimicrobial effect is more expressed than that of penicillin, biomycin and other antibiotics, due to the inhibitory effect on antibiotic-resistant strains of bacteria. According to the data of these works, the effect of killing bacteria with preparations containing elemental silver nanoparticles is 1.500 times higher than phenol at the same concentration, and 3.5 times higher than mercury(II) dichloride (with much less toxicity). Ag-NP have an antimicrobial effect on many pathogenic microorganisms, such as *Staphylococcus aureus*, *Streptococcus aureus*, *Proteus vulgaris*, *Pseudomonas aeruginosa* and *Escherichia coli*: from bacteriostatic (ability to inhibit microbial reproduction) to bactericidal (ability to destroy microbes) [8]. Currently published works describe the use of biosynthesized Ag-NPs as antibacterial agents against a wide variety of microorganisms. So, in a number of works cited above devoted to the “green synthesis” of Ag-NP with the participation of plant extracts, a high efficiency of silver nanoparticles against pathogenic microorganisms of genera *Bacillus* [43,73,74,81,87,103,106], *Staphylococcus* [36,40,43,47,56,69,74,81,85,87,90,93,100,103,106,108], *Pseudomonas* [40,43,47,69,77,85,93,100,103], *Klebsiella* [41,69,77,85,87,93,100,106], *Escherichia* [36,56,69,73,74,77,81,83,85,87,90,93,100,103,108], *Salmonella* [74,77], *Enterococcus* [43] and *Serratia* [81], was noted. A similar Ag-NP effect received by various microorganisms and microscopic fungi on the genera *Bacillus* [133,155,162,186], *Staphylococcus* [138,140,146,147,155,162,176,186], *Pseudomonas* [138,146,155], *Klebsiella* [176], *Escherichia* [133,155,162,176,186], *Salmonella* [138,140,147,155,162,176,186], *Enterococcus* [138] and *Streptococcus* [140] was observed. The introduction of Ag-NP causes structural and morphological changes in cells that can lead to bacterial death. When silver nanoparticles come into contact with bacteria, they adhere to the cell wall and cell membrane, prevent replication and contribute to cell death [239]. Meanwhile, the so-called electronic effects are observed for Ag-NP with an average size of 10 nm or less, as a result of which their bactericidal activity increases sharply compared to that for Ag-NP with large dimensions [240]. In quantitative ratio, this effect is different for each specific type of cell, since, on the one hand, the composition of their cell membranes varies widely, on the other hand, with a decrease in the size of Ag-NP, their reactivity increases due to an increase in their surface area and reduce their volume. According to work [108] the discovered silver nano-wedges, due to their unique pointed shape, act on any microorganisms like real “daggers”, tearing their bodies apart. Therefore, such Ag-NP with a similar form may be promising candidates for wide range of biomedical applications, and especially in the manufacture of antibacterial drugs. High fungicidal activity of biosynthesized Ag-NP on some microscopic fungi [108,119,126,139] was shown. But it is noteworthy that, apparently, there are no works devoted to the biosynthesis of Ag-NP with the participation of micro-fungi in which the authors pointed to the fungicidal activity of the obtained Ag-NP to the micro-fungi, by which these nanoparticles were synthesized.

An important fact is that clearly expressed anticancer activity of silver nanoparticles was identified [52,54,61,70,73,79,87,88,101,156,162]. In [241], and Ag-NP were proposed for cancer diagnosis and drug standards. In [242], chemotherapeutic anticancer drugs were developed with photo-soluble linkers that "attached" them to a substrate on the surface of Ag-NP. The principle of action of such drugs is reduced to destruction under the influence of UV radiation, resulting in its active form, which has a destructive effect on cancer cells [242]. Earlier, an alternative approach, in which the anticancer drug “attached” directly to the functionalized surface of Ag-NP, was proposed [243]. The advantages of cancer treatment methods described in [242,243] are that, on the one hand, the drug is transported into the patient’s body without the use of any toxic compounds, on the other hand, it is selectively released precisely in the affected organ. Ag-NP can also be useful for overcoming multidrug resistance, which often prevents the delivery of the right drug to the affected organ [244].

Ag-NP seems to be characterized by a highly synergistic bactericidal action in combination with such well-known antibiotics as penicillin, ampicillin, erythromycin, clindamycin and vancomycin; such a phenomenon has been observed, for example, against bacteria of the genus *Staphylococcus* and *Escherichia* [245]. It should be noted that preparations based on elemental nanoparticles are widely used in bone transplants for the treatment of burns, because Ag-NP associated with the implant provide better antimicrobial activity and contribute to a significant reduction in the number of scars arising in the healing process of the affected tissue. Owing to antimicrobial activity, elemental silver nanoparticles find a certain application also in the food industry and in food technologies, described in [246,247]. 

Another possibility for the application Ag-NP is their use as part of larvicidal compositions. In particular, it was proposed to use biosynthesized Ag-NP against malaria mosquitoes of the genus *Anopheles* [33,105,248,249,250,251,252,253], and pathogens of malaria, *Plasmodium falciparum* [254], and mosquito carriers of yellow fever, the genus *Aedes* and *Culex* [104,105,106,248,249,251,252,253,255]. (It is interesting that graphene was used in [251,254] as one of the components of such preparations). However, the number of works devoted to the larvicidal (and insecticidal) activity of biosynthesized Ag-NP is still relatively small compared to that for the works about the antibacterial activity of these NP.

Nevertheless, it should be noted that Ag-NP is still toxic to the human body. Because of Ag-NP dissolves to form Ag^+^ ions, which are known to have toxic effects [256], some studies have been conducted to determine whether Ag-NP toxicity is a result of the release of silver ions or is associated with the nanoparticles themselves [257,258]. The results of these studies indicate that elemental silver nanoparticles can indeed cause allergies. However, these results do not exclude the possibility that the toxicity of Ag-NP is no less associated with the formation of silver ions in cells, because according to [259], Ag-NP and Ag^+^ ions have almost the same cytotoxicity. The authors of [260] came to the same conclusion: the combination of Ag-NP and Ag^+^ is responsible for the toxic effect of silver nanoparticles; in addition, a toxic effect on cells was shown for the Ag-NP regardless of free silver ions. On the other hand, the toxicity of Ag-NP in human cells is due to oxidative stress and inflammation caused by the formation of reactive oxygen species stimulated by either Ag-NP, Ag^+^ ions, or both [261]. According to the authors of [262], the introduction of Ag-NP into tissue cells leads to the formation of free radicals, which pose a potential health risk.

## 7. Conclusions

Thus, the perspectives of the biosynthesis of Ag-NP look very impressive. The list of biological substrates that have so far been used in published works is extremely long, and in this review paper it is impossible even to quote all these publications. However, now the development and improvement of these methods using those discussed biological substrates, the control of the size, shape and degree of dispersion of biosynthesized Ag-NP, cannot yet be considered to have been adequately determined experimentally. That is why, for the implementation of the controlled biosynthesis of Ag-NP with predetermined target parameters, undoubtedly, some new principles and methodological approaches should be elaborated. For this, a thorough knowledge of the mechanism of the process of nanoparticle biosynthesis is necessary in general and of Ag-NP in particular, the specifics of which in most cases has remained unexplored. Each of these bio-syntheses requires information about the effect on the process of concentration-time and temperature parameters used in the experiment, which in most published works is scattered and clearly insufficient to make complete conclusions. A very important task is also to increase the yield of the target product (i.e., synthesized Ag-NP). Finally, it is very important to improve existing methods and develop new methods of isolating nanoparticles from the parent systems in which they were formed (which may be necessary to produce commercially available products containing these nanoparticles). 

## Figures and Tables

**Table 1:** Sizes and shapes of Ag-NP obtained by “green synthesis” using plant extracts.

Plant	Part Used for Synthesis	Shape (form)	Size of Ag-NP (nm)	Max of Absorption of Ag-NP in Visible Spectrum (nm)	Ref.
*Pelargonium graveolens* (Geranium)	Leaves	Spherical	16–40	440	[25]
*Emblica Officinalis* (Amla, Indian Gooseberry)	Fruits	Spherical	15–25	400–420	[26]
*Aloe Vera*	Leaves	Spherical	15.2 ± 4.2	410	[27]
*Capsicum annuum*	Leaves	Spherical	50–70	428	[28]
*Lippia citriodora* *(Lemon Verbena)*	Leaves	Spherical	15–30	430–440	[29]
*Acalypha indica*	Leaves	Spherical	20–30	425	[30]
*Tribulus terrestris*	Exsiccated leaves	Spherical	18–47	450	[31]
*Mimusops elengi*	Leaves	Spherical	55–83	440	[32]
*Chrysanthemum indicum*	Flowers	Spherical	25–59	430	[33]
*Cinnamomum canphora*	Leaves	Spherical	55–80	440	[34]
*Eclipta*	Leaves	Spherical	2–6	419	[35]
*Ocimum sanctum* (Tulsi)	Leaves	Spherical	4–30	413	[36]
*Cassia auriculata*	Leaves	Spherical	1–100	450	[37]
*Euphorbia hirta* *Nerium indicum*	Leaves	Spherical	29–31	380, 460	[38]
*Rosa Chinensis*	Leaves	Spherical	25–60	No λmax data	[39]
*Tribulus terrestris*	Fruit	Spherical	16–28	435	[40]
*Dioscorea bulbifera*	Tuber	Triangular, Nanorod	8–20	455	[41]
*Ulva lactucain*	Whole plant	Spherical	76	434	[42]
*Trianthema decandra*	Root	Spherical	36–74	Absent	[43]
*Cissus quadrangularis*	Whole plant	Spherical	50–100	450	[44]
*Iresine herbstii*	Leaves	Spherical	44–64	420	[45]
*Ananas comosus*	Fruits	Spherical	~12	430	[46]
*Boswellia serrata*	Gum	Spherical	7.5 ± 3.8	420	[47]
*Hibiscus cannabinus*	Leaves	Spherical	9–10	446	[48]
*Piper pedicellatum*	Leaves	Spherical	2–30	440	[49]
*Tithonia diversifolia*	Leaves	Spherical	~25	Absent	[50]
*Ficus panda*	Leaves	Spherical	12–36	421	[51]
*Citrullus colocynthis*	Leaves Root Seeds	Spherical Spherical Spherical	13.37 7.39 16.57	No. λmax data	[52]
*Alternanthera sessilis*	Leaves	Spherical	30–50	420	[53]
*Podophyllum hexandrum*	Leaves	Spherical	~14	430	[54]
*Cocos nucifera*	Inflorescence	Spherical	~22	420	[55]
*Olea europaea* (Olive)	Leaves	Spherical	20–25	441–456	[56]
*Sargassum wightii* (algae)	Whole	Spherical	5–22	439	[57]
*Olax scandens*	Leaves	Spherical	30–60	410–430	[58]
*Piper longum*	Fruits	Spherical	~46	465	[59]
*Delonix elata*	Leaves	Spherical	35–45	432	[60]
*Adansonia digitata*	Fruits	Spherical	3–57	434	[61]
*Emblica officinalis*	Fruits	Spherical	15–20	425	[62]
*Rheum emodi*	Root	Spherical	10–40	425	[63]
*Allium sativum*	Whole plant	Spherical	100–800	No. λmax data	[64]
*Sargassum vulgare* (algae)	Whole plant	Spherical	~10	No. λmax data	[65]
*Erythrina indica* lam	Root	Spherical	20–118	438	[66]
*Plumeria alba*	Flowers	Spherical	36.2	455	[67]
*Cymodocea serrulata*	Whole plant	Spherical	17–29	430	[68]
*Skimmia laureola*	Leaves	Spherical		460	[69]
*Butea monosperma*	Leaves	Spherical	20–80	440–475	[70]
*Capparis decidua*	Leaves	Spherical	1.5–25	452	[71]
*Azadirachta indica*	Leaves	Spherical	~34	436–446	[72]
*Syzygium cumini* *Azadirachta indica*	Flowers Leaves	Spherical Spherical	<40 <40	400–450	[73]
*Capparis spinosa*	Leaves	Spherical	10–40	420	[74]
*Cola nitida*	Pods	Spherical	12–80	431	[75]
*Artemisia marschalliana*	Aerial part	Spherical	5–50	430	[76]
*Ziziphus oenoplia*	Leaves	Spherical	10	436	[77]
*Croton bonplandianum Baill.*	Leaves	Spherical	32	425	[78]
*Dimocarpus longan*	Peel	Spherical	8–22	No. λmax data	[79]
*Rubus glaucus*	Leaves	Spherical	12–50	440–445	[80]
*Raphanus sativus*	Leaves	Spherical	4–30	426	[81]
*Melia azedarach*	Leaves	Spherical	34–48	482	[82]
*Calliandra haematocephala*	Leaves	Spherical	13.5–91.3	414	[83]
*Crocus sativus*	Leaves	Spherical	12–20	450	[84]
*Costus afer*	Leaves	Spherical	~20	405–411	[85]
*Punica granatum*	Peel	Spherical	20–40	Absent	[86]
*Cleome viscosa*	Fruits	Spherical	20–50	410–430	[87]
*Anthemis atropatana*	Aerial part	Spherical	10–80	430	[88]
*Citrullus colocynthis*	Callus	Spherical	~31	No. λmax data	[89]
*Datura stramonium*	Leaves	Spherical	15–20	444	[90]
*Morinda citrifolia*	Root	Spherical	30–55	413	[91]
*Ficus talboti*	Leaves	Spherical	10–14	438	[92]
*Potentilla fulgens*	Root	Spherical	10–15	410	[93]
*Syzygium cumini* *Citrus sinensis* *Solanum tricobatum* *Centella asiatica*	Leaves powder	Triangular	53 41 52 42	420	[94]
*Rheum palmatum*	Root	Hexagonal	121 ± 2	440	[95]
*Alysicarpus monilifer*	Leaves	Hexagonal, Spherical	5–45	422	[96]
*Melia azedarach*	Leaves	Cubic	78	436	[97]
*Eucalyptus macrocarpa*	Leaves	Cubic	10–50	430	[98]
*Cucurbita maxima* *Moringa oleifera* *Acorus calamus*	Petals Leaves Rhizome	Cubic	30–70	Absent	[99]
*Ocimum tenuiflorum* *Solanum tricobatum* *Syzygium cumini* *Centella asiatica* *Citrus sinensis*	Leaves	Prismatic	28 22.3 26.5 28.4 65	420 420 420 415 415	[100]
*Achillea biebersteinii*	Flowers	Pentagonal Spherical	10–40	450	[101]
*Solanum trilobatum*	Fruits	Polygonal	41–42	420	[102]
*Musa paradisiaca* (banana)	Peels	Irregular	~24	433	[103]
*Annona squamosa*	Leaves	Irregular	~300	420	[104]
*Artemisia nilagirica*	Leaves	Irregular	≤30	463	[105]
*Tinospora cordifolia*	Leaves	Irregular Spherical	~30	430	[106]
*Leucas aspera* *Hyptis suaveolens*	Leaves Leaves	Irregular Polygonal	7–22 5–25	401 408	[107]
*Órchis máscula*	Tuber	“Flower-like”	<100 (width) ~500 (length)	444	[108]

**Table 2:** Sizes and shapes of Ag-NP received by “green synthesis” by using various microorganisms.

Microorganism (Type)	Shape (form)	Size of Ag-NP (nm)	Max of Absorption of Ag-NP in Visible Spectrum (nm)	Ref.
*Pseudomonas stutzeri* (bacteria)	Spherical Triangular Hexagonal	70–200	400	[115]
*Pseudomonas stutzeri* (bacteria)	Spherical Triangular Hexagonal	70–200	400	[116]
MKY3 strain (bacteria)	Spherical Hexagonal	~26	420	[117]
*Fusarium oxysporum* (fungus)	Spherical	5–15	413	[118]
*Fusarium oxysporum* (fungus)	Spherical	20–50	420	[119]
*Aspergillus flavus* (fungus)	Spherical	~9	420	[120]
*Fusarium acuminatum* (fungus)	Spherical	5–40	420	[121]
*Bacillus licheniformis* (bacteria)	Spherical	~40	Absent	[122]
*Bacillus licheniformis* (bacteria)	Spherical	~50	440	[123]
*Escherichia coli* (bacteria)	Spherical	~50	420	[124]
*Klebsiella pneumonia* (bacteria)	Spherical	1–6	420	[125]
*Aspergillus niger* (fungus)	Spherical	3–30	430	[126]
*Brevibacterium casei* (bacteria)	Spherical	10–50	420	[127]
*Pseudomonas aeruginosa* (bacteria)	Spherical	~13	430	[128]
*Rhizopus stolonifer* (fungus)	Spherical	3–20	Absent	[129]
*Pseudomonas antarctica* (bacteria) *Pseudomonas proteolytica* (bacteria) *Pseudomonas meridian* (bacteria) *Arthrobacter kerguelensis*(bacteria) *Arthrobacter gangotriensis* (bacteria) *Bacillus indicus* (bacteria) *Bacillus cecembensis* (bacteria)	Spherical	6–13	400–430	[130]
*Penicillium purpurogenum* (fungus)	Spherical	8–10	390–420	[131]
*Bacillus subtilis* (bacteria)	Spherical Triangular Hexagonal	45–70	440	[132]
*Bacillus amyloliquefaciens* (bacteria)	Spherical Triangular	~15	420–425	[133]
*Streptomyces sp*. (bacteria)	Spherical	21–48	441	[134]
*Streptomyces albogriseolus* (bacteria)	Spherical	16.25 ± 1.6	409	[135]
*Salmonella typhirium* (bacteria)	Spherical Ellipsoidal	87 ± 30	427	[136]
*Pencillium sp*. (fungus)	Spherical	25	425	[137]
*Acinetobacter calcoaceticus* (bacteria)	Spherical	8–60	420–440	[138]
*Aspergillus fumigatus* (fungus)	Spherical	20–140	420	[139]
*Bacillus subtilis* (bacteria)	Spherical	No data	420	[140]
*Streptomyces sp.* (bacteria)	Spherical	50–86	420	[141]
*Penicillium sp*. (fungus)	Spherical	25–30	420	[142]
*Bacillus sp.* (bacteria)	Spherical	42–94	450	[143]
*Actinomycetes* (bacteria)	Spherical	10–20	415	[144]
*Penicillium glabrum* (fungus)	Spherical	26–32	420	[145]
*Streptomyces sp.* (bacteria)	Spherical	50–76	420	[146]
*Ochrobactrum sp*. (bacteria)	Spherical	38–85	450	[147]
*Fusarium oxysporum* (fungus)	Spherical	15–40	420	[148]
*Penicillium atramentosum* (fungus)	Spherical	5–25	420	[149]
*Variovorax guangxiensis* (bacteria)	Spherical	10–40	418	[150]
*Sporosarcina koreensis* (bacteria)	Spherical	10–30	424	[151]
*Penicillium brevicompactum* (fungus)	Spherical	30–50	420	[152]
*Pseudomonas deceptionensis* (bacteria)	Spherical	10–30	428	[153]
*Bacillus methylotrophicus* (bacteria)	Spherical	10–30	416	[154]
*Streptomyces rochei* (bacteria)	Almost ideally spherical	22–85	410	[155]
*Streptomyces atrovirens* (bacteria)	Spherical	58 ± 2	418	[156]
*Rhizopus stolonifer* (fungus)	Spherical	3–50	420	[157]
*Aeromonas sp*. (bacteria)	Spherical	8–16	400	[158]
*Bacillus brevis* (bacteria)	Spherical	41–68	420	[159]
*Phenerochaete chrysosporium* (bacteria)	Spherical	34–90	430	[160]
*Streptacidiphilus durhamensis* (bacteria)	Spherical	8–48	430	[161]
*Penicillium italicum* (fungus)	Spherical	14.5–23.3	423	[162]
*Streptomyces xinghaiensis* (bacteria)	Spherical	5–20	420	[163]
*Enterobacter cloacae* (bacteria)	Spherical	7–25	440	[164]
*Streptomyces olivaceus* (bacteria)	Spherical	~12.3	450	[165]
*Paracoccus sp*. (bacteria)	Spherical Ellipsoidal	2–5	416	[166]
*Aspergillus fumigates* (fungus)	Irregular	5–25	420	[167]
*Aspergillus clavatus* (fungus)	Irregular	550–650	420	[168]
*Bacillus megaterium* (bacteria)	Irregular	80–99	Absent	[169]
*Aspergillus flavus* (fungus)	Irregular	17 ± 5.9	421	[170]
*Pseudomonas aeruginosa* (bacteria)	Irregular	2–20	425	[171]
*Idiomarina sp* (bacteria)	Irregular	26	450	[172]
*Staphylococcus aureus* (bacteria)	Irregular	28–50	420–430, 550–570	[173]
*Streptomyces sp*. (bacteria)	Irregular	68	423	[174]
*Enterococcus sp.* (bacteria)	Irregular	30–100	Absent	[175]
*Streptomyces sp*. (bacteria)	Irregular	70–100	400	[176]
*Acinetobacter baumannii* (bacteria)	Irregular	37–168	Absent	[177]
*Pseudomonas sp*. (bacteria)	Irregular	10–40	412	[178]
*Bacillus flexus* (bacteria)	Triangular	12–65	420	[179]
*Bacillus stratosphericus* (bacteria)	Triangular Hexagonal Cubic	2–20	405	[180]
*Fusarium semitectum* (fungus)	Hexagonal Spherical	10–60	420	[181]
*Aspergillus clavatus* (fungus)	Hexagonal Spherical	10–25	415	[182]
*Bacillus licheniformis* (bacteria)	Hexagonal Triangular	22–44	422	[183]
*Streptomyces viridodiastaticus* (bacteria)	Polygonal	15–45	400	[184]
*Arthrospira maxima* (cyanobacteria) *Arthrospira platensis* (cyanobacteria) *Hapalosiphon fontinalis* (cyanobacteria) *Spirulina sp.* (cyanobacteria) *Cylindrospermum stagnale* (cyanobacteria) *Spirulina sp.* (cyanobacteria) *Phormidium sp.* (cyanobacteria) *Spirulina sp.* (cyanobacteria) *Calothrix brevissema* (cyanobacteria)	Triangular Triangular Triangular Pentagonal Pentagonal Hexagonal Cubic Cubic Cubic	61 46 50 51 38–40 47 48 49 42	465 445 450 450 440 446 446 450 443	[185]
*Hargavaea indica* (bacteria)	Pentagonal Spherical Icosahedral Hexagonal Triangular Icosahedral Truncated triangle	30–100	460	[186]
